# Mathematical modeling of the effect of early detection in breast cancer treatment

**DOI:** 10.3389/fonc.2025.1626435

**Published:** 2025-09-08

**Authors:** Farouk Tijjani Saad, Yusuf Ya’u Gambo, Phollakrit Wongsantisuk, Idris Ahmed, Jessada Tariboon

**Affiliations:** ^1^ Department of Mathematics, Northwest University, Kano, Nigeria; ^2^ Department of Electronics Engineering Technology, College of Industrial Technology, King Mongkut’s University of Technology North Bangkok, Bangkok, Thailand; ^3^ Department of Mathematics, Faculty of Natural and Applied Sciences, Sule Lamido University, Kafin Hausa, Jigawa, Nigeria; ^4^ Intelligent and Nonlinear Dynamics Innovation Research Center, Department of Mathematics, Faculty of Applied Science, King Mongkut’s University of Technology North Bangkok, Bangkok, Thailand

**Keywords:** breast cancer, modeling, sensitivity analysis, early detection, immune response, awareness

## Abstract

**Introduction:**

Early detection is a cornerstone of cancer control, yet its quantitative influence on tumor–immune interactions remains underexplored in mathematical oncology.

**Methods:**

We formulated a nonlinear tumor–immune interaction model consisting of two coupled ordinary differential equations for tumor growth and immune response. Early detection was represented by a saturating function (Michealis-Menten term) dependent on tumor size and awareness level. The system was nondimensionalized to reduce parameters and ease analysis. Equilibria were derived, and both local and global stability were analyzed. Numerical simulations, phase portraits, bifurcation and sensitivity analyses were conducted to assess system behavior and parameter influence.

**Results:**

Two biologically meaningful equilibria were identified. Stability analyses established the conditions for sustained tumor-free states. Simulations demonstrated that higher awareness significantly enhances early detection, thereby suppressing tumor growth. Phase portraits revealed stable tumor–immune dynamics, while sensitivity results highlighted awareness- and detection-related parameters as the most critical for tumor control.

**Discussion:**

The model quantifies the role of awareness-driven early detection in shaping tumor–immune outcomes. Results underscore the importance of public awareness campaigns, screening initiatives, and early intervention strategies for effective cancer management. This framework bridges mathematical modeling and policy, offering understanding into optimizing awareness-based control measures.

## 1 Introduction

Breast cancer remains one of the most challenging and predominant malignancies worldwide, requiring innovative ways to understand its progression and improve early detection schemes (1, 2). It initiates with genetic mutations, which lead to uncontrolled cell growth, eventually forming a tumor inside the breast. This evolution involves various stages, which include hyperplasia, carcinoma *in situ*, invasive carcinoma, and metastasis (3, 4). Recent studies have highlighted the role of cancer stem cells in tumor origination and development (5–7).

Early detection methods, such as mammography, biomarker analysis, and MRI, are critical for detecting breast cancer at its most treatable stage. Additionally, a large-scale study comparing the effectiveness of digital and film mammography in early breast cancer detection found that digital mammography is particularly beneficial for younger women and for those with dense breast tissue (8). An individual may notice signs or symptoms of breast cancer under standard care, prompting them to seek for medical evaluation and eventually receiving a proper diagnosis. In contrast, earlier detection occurs when an individual without any noticeable signs or symptoms is diagnosed through a specialized screening examination (9). Recent evolving technologies, such as AI-assisted imaging and liquid biopsies, show potential in improving the accuracy of early detection (10).

Lee and Zelen (2003) studied a stochastic model for predicting breast cancer mortality, focusing on the effects of early detection and treatment. The model estimates survival probabilities based on tumor progression and the timing of diagnosis, providing insights into the impact of screening programs on breast cancer outcomes. It further found that early detection of breast cancer through screening significantly reduces mortality by identifying tumors at a more treatable stage (9, 10).

The immune system plays a crucial role in recognizing and eliminating cancer cells (11). Schreiber et al. (12) reviewed the mechanisms of cancer immunoediting, demonstrating how the immune system can both suppress and promote tumor growth, and highlighting the importance of a balanced immune response, where key players include cytotoxic T lymphocytes (CTLs), natural killer (NK) cells, and macrophages. Recent research has also focused on the role of regulatory T cells and myeloid-derived suppressor cells in modulating the antitumor immune response (13, 14).

Mathematical modeling has emerged as a powerful tool in oncology, offering insights into tumor growth dynamics and the complex interactions between cancer cells and the immune system (11, 14–22). The application of mathematical models to cancer research has a rich history, dating back to the seminal work of Norton and Simon in 1977, who proposed a Gompertzian model of tumor growth (23). Since then, numerous models have been developed to capture various aspects of cancer biology, including the pivotal role of the immune system in tumor suppression and progression (23, 24). Recent advancements in early detection technologies have significantly improved breast cancer outcomes. However, the precise impact of early detection on tumor–immune dynamics remains incompletely understood.

Mathematical modeling offers a unique opportunity to explore these interactions and potentially optimize screening protocols (25). These models range from simple ordinary differential equations to complex agent-based models and have been instrumental in predicting treatment outcomes and optimizing therapy schedules (26). The study in (26) emphasized the importance of collaboration between mathematicians, biologists, and clinicians in developing and validating predictive models for cancer treatment. While optimal control theory provides a framework for personalized treatment, more research is needed to develop and validate personalized models that account for individual patient variability.

Optimal control theory involves determining the best intervention strategies to achieve desired outcomes, such as minimizing tumor size while maximizing immune response. Lenhart (27) applied optimal control theory to biological models, illustrating how this approach can be used to design optimal treatment schedules for cancer patients, balancing efficacy and side effects. This approach has been applied to various cancer treatment modalities, including chemotherapy, immunotherapy, and radiotherapy (28). The study in (29) explores the interactions between tumor cells and the immune system, including immune surveillance, immune evasion, and immunoediting.

Recent advances in this field have led to the development of the cancer immunogram, a framework for understanding the multifaceted nature of tumor–immune interactions (30). Stability analysis involves examining the equilibrium points of the ODE system to understand the conditions under which the system remains stable or undergoes changes, such as tumor regression or progression (31–34). Advanced techniques, such as bifurcation analysis, have been applied to study the qualitative changes in system behavior under varying parameters (34–36).

More recently (37), analyzed long-term trends in breast cancer incidence, concluding that improved screening has led to increased detection of early-stage cancers but has had a limited impact on the incidence of advanced disease. Building on the work of Schreiber et al. (38), conducted a comprehensive analysis of the tumor microenvironment, revealing the prognostic significance of various immune cell subsets in different cancer types.

Recently (39), discussed the integration of mathematical modeling with clinical data to develop personalized treatment approaches in oncology. Building on this foundation, Sharifi et al. developed an optimal control framework for combination immunotherapy, demonstrating how mathematical modeling can guide the design of more effective treatment protocols (40).

Combining early detection methods with an understanding of the immune response provides a comprehensive strategy for managing breast cancer. Early detection is critical for identifying treatable stages of the disease, while the immune response plays a significant role in controlling tumor growth. Recent studies, such as those by (41), have highlighted the potential of using immune biomarkers in conjunction with traditional screening methods to improve early detection and prognosis prediction.

Modeling and optimal control theory offer a structured approach to determining the best intervention strategies, balancing efficacy and safety. This approach can lead to personalized treatment plans that maximize patient outcomes (42–44). Recent work by (45) has demonstrated the potential of using optimal control theory to design adaptive therapy protocols that may delay or prevent the onset of drug resistance in cancer treatment. Recent advances in single-cell sequencing technologies offer new opportunities to characterize tumor heterogeneity and develop more precise models (41, 45).

Advancing the field requires collaboration between oncologists, immunologists, mathematicians, and data scientists. Such interdisciplinary efforts can lead to the development of more sophisticated models and effective treatment strategies. Initiatives like the Physical Sciences Oncology Network (PSON) are fostering such collaborations, but more work is needed to bridge the gap between theoretical models and clinical practice (46). While many mathematical models show promise in simulating cancer dynamics, there is a need for rigorous validation using clinical data.

The work of (39) provides a framework for integrating mathematical modeling into clinical trials, but wider adoption of these approaches is needed. As our understanding of cancer biology evolves, mathematical models need to be updated to incorporate new insights. For example, recent discoveries about the role of the microbiome in cancer progression and treatment response by (46) present new challenges and opportunities for model development. As models become more complex, incorporating multiple scales and high-dimensional data, there is a need for advanced computational methods to solve and analyze these models efficiently (42).

Mathematical modeling has played a vital role in understanding the progression and treatment of breast cancer. Several studies have proposed models capturing key aspects of tumor dynamics, treatment response, and microenvironmental interactions (39, 42).

Benzekry et al. developed a mathematical model using systems of ordinary differential equations to explore and investigate systemic interactions between primary and metastatic tumors, focusing particularly on breast cancer. Their work demonstrated that distant tumors could exert inhibitory or stimulatory effects on each other’s growth. This suggests the need for a systemic view of tumor control beyond localized therapy (47). The role of cancer stem cells in breast cancer response to radiotherapy, using a spatial mathematical model, was examined in (48), highlighting the significance of the spatial distribution of stem-like and proliferative cells within a tumor. Their findings showed that treatment outcomes were not only dependent on the quantity of cancer cells but also on their spatial organization and phenotypic heterogeneity.

Pérez-García et al. (49) proposed a nonlinear mathematical model to analyze the combined effects of chemotherapy and radiotherapy on tumor growth. Although the model is not limited to breast cancer, it is generalizable and applicable to solid tumors. The study generally offered insights into the design of optimal treatment schedules and conditions for tumor eradication, underscoring the impact of treatment timing and intensity on tumor dynamics.

An agent-based modeling (ABM) approach is now being employed to simulate the progression of ductal carcinoma *in situ* (DCIS), a common form of early-stage breast cancer (50). Their multi-scale model integrated cellular behaviors with tissue-level structures, enabling the study of spatial tumor growth, intraductal invasion, and treatment effects. This work emphasized the utility of *in silico* platforms in understanding the heterogeneity and morphology of early breast lesions. Macklin and Lowengrub focused on the influence of the tumor microenvironment on breast cancer development. They introduced a hybrid model that coupled mechanical pressure, nutrient diffusion, and extracellular matrix interactions. Their simulations revealed that tumor shape and invasion patterns were significantly affected by microenvironmental conditions, demonstrating the importance of spatial and physical factors in tumor progression (51).

More recent efforts have extended these frameworks using advanced methodologies. Dołęga-Kozierowski et al. integrated numerical modeling with AI and image fusion to produce personalized 3D breast cancer models for enhanced prediction of tumor growth and spatial behavior (52). A virtual twin computational model that predicts neoadjuvant therapy outcomes in individual patients based on imaging and clinical data was introduced in (53). These studies illustrate a growing trend toward data-driven, patient-specific modeling. Li and Thirumalai presented a model focusing on phenotypic heterogeneity, particularly HER2 status, and showed how this influences treatment sequencing and resistance (54). In a related study, Yang et al. developed a model to track breast cancer cell population dynamics under doxorubicin exposure, quantifying drug resistance at the cellular level (55). Idrees et al. applied fractal–fractional calculus to breast cancer dynamics, enabling more accurate depiction of memory and nonlocal effects in tumor growth (15).

Recent advances in early detection and tumor–immune interaction modeling have highlighted the importance of integrating biological data and modern computational tools to improve breast cancer diagnosis and treatment outcomes. Zeng et al. (56) demonstrated the potential of combining serum Raman spectroscopy with convolutional neural networks for rapid and accurate identification of breast cancer subtypes, providing a powerful tool for early detection. Pang et al. (57) investigated the prognostic significance of systemic immune-inflammation indices in HER2-positive metastatic breast cancer, emphasizing the critical role of immune markers in clinical decision-making. Similarly, Liu, Zhang, and Zhao (58) identified transmembrane protein 100 as a biomarker for malignant progression and chemosensitivity, underscoring the molecular basis for individualized treatment strategies.

From an immunotherapy perspective, Zhu et al. analyzed the efficacy of PD-1 inhibitor therapy combined with the GP regimen in advanced triple-negative breast cancer, reflecting current trends in combining immune checkpoint blockade with chemotherapy (59). In addition, Cui et al. (60) developed a nomogram to predict radiation-induced dermatitis, aiding in personalized radiation treatment planning. Advances in artificial intelligence have also contributed to model-informed oncology, where it is applied as causal representation learning for radiology report generation, bridging imaging and clinical semantics in cancer care (61).

Despite the richness of existing models, most do not explicitly incorporate the role of early detection and public awareness in controlling tumor growth. This is a crucial gap given the known benefits of early diagnosis in reducing breast cancer mortality. The model proposed in the present study addresses this by introducing an awareness-driven early detection mechanism, mathematically embedded into the tumor growth dynamics. This novel approach allows for quantitative evaluation of how awareness campaigns, screening programs, and public health interventions can influence cancer outcomes—an area not sufficiently explored in previous literature.

We adopt the mathematical model in (17) and incorporate an awareness term that signifies early detection of breast cancer to stress the importance and effect of early detection in the treatment of breast cancer. The motivation for this research is driven by the urgent need to enhance early detection methods and treatment strategies for breast cancer. By bridging the gap between theoretical modeling and clinical practice, this study aims to provide valuable tools and insights that can inform both researchers and healthcare professionals in their efforts to reduce breast cancer mortality and improve the quality of life for patients.

This study is organized as follows: *Section 1* provides the introduction. The model is presented in *Section 2*, while *Section 3* deals with the stability and sensitivity analysis of the model. We introduce the optimal control problem and its analysis in *Section 4*. We present the numerical simulations in the last section, followed by the conclusion and discussion.

## 2 Presentation of the model

The model in (17) is given in (Equation 1) as follows:


(1) $$\begin{array}{l} \frac{dC\left(t\right)}{dt}=a_{1}C\left(t\right)\left(1-\frac{C\left(t\right)}{a_{2}}\right)-a_{3}C\left(t\right)\left(\frac{L\left(t\right)}{a_{4}+L\left(t\right)}\right),\\ \frac{dL\left(t\right)}{dt}=a_{5}L\left(t\right)\left(1-\frac{L\left(t\right)}{a_{6}}\right)\left(\frac{C\left(t\right)}{a_{7}+C\left(t\right)}\right)-a_{8}C\left(t\right)L\left(t\right)-a_{9}L\left(t\right), \end{array}$$

Where C(t) represents the population of breast cancer cells at time t , L(t) represents the population of cytotoxic T lymphocytes, $a_{1}$ is the tumor intrinsic growth rate, $a_{2}$ is the carrying capacity for breast cancer cells, $a_{3}$ represents the rate at which tumor cells are killed by lymphocytes, $a_{4}$ is the saturation constant for the killing of tumor cells by lymphocytes, $a_{5}$ is proliferation rate of lymphocytes, $a_{6}\;$ is carrying capacity for lymphocytes, $a_{7}$ represents saturation constant for lymphocyte proliferation, $a_{8}\;$ is the rate of lymphocyte death due to cancer cells, and $a_{9}$ is the natural death rate of lymphocytes due to apoptosis. Our mathematical model given in (Equation 2) is an extension of (17) by incorporating the early detection term as follows:


(2) $$\begin{array}{l} \frac{dC\left(t\right)}{dt}=a_{1}C\left(t\right)\left(1-\frac{C\left(t\right)}{a_{2}}\right)-a_{3}C\left(t\right)\left(\frac{L\left(t\right)}{a_{4}+L\left(t\right)}\right)-f\left(C\right),\\ \frac{dL\left(t\right)}{dt}=a_{5}L\left(t\right)\left(1-\frac{L\left(t\right)}{a_{6}}\right)\left(\frac{C\left(t\right)}{a_{7}+C\left(t\right)}\right)-a_{8}C\left(t\right)L\left(t\right)-a_{9}L\left(t\right), \end{array}$$

Where f(C) is the effect of early detection, which is a function of C,  the tumor size. This effect is influenced by tumor size C(t) , detection threshold $\tilde{C}$ , and awareness parameter α. The function f(C) expressed in (Equation 3) is considered to follow the Michaelis–Menten term (62–66). Thus, it is given as follows:


(3) $$f\left(C\right)=\frac{KC\left(t\right)}{a+\alpha \tilde{C}C\left(t\right)},$$

where K represents the maximum effect of early detection, C(t) is the tumor size at time $t,\;\tilde{C}$ represents the tumor size threshold for detection, a is a constant that ensures the function behaves properly for small C(t) by providing a baseline detection effectiveness, and α is the awareness parameter. We can observe that high awareness (α) makes early detection more effective, lowering the overall impact of tumor size. Conversely, low awareness makes early detection less effective, allowing the tumor to grow more before detection has a significant effect. This term ensures that as C(t) grows, detection saturates, reflecting that detection effectiveness reaches a maximum point.

For ease of analysis, we nondimensionalize our model to reduce the number of parameters involved. The nondimensionalized model is given by:


(4) $$\begin{array}{l} \frac{dx}{d\tau }=x\left(1-x\right)-\beta x\left(\frac{y}{\gamma +y}\right)-\frac{\kappa x}{\alpha +\delta x},\\ \frac{dy}{dt}=\rho y\left(1-y\right)\left(\frac{x}{\mu +x}\right)-\sigma xy-\eta y, \end{array}$$

where,


$$\begin{array}{c} x=\frac{C}{a_{2}},\;y=\frac{L}{a_{6}},\;\tau =a_{1}t,\;\beta =\frac{a_{3}a_{6}}{a_{1}a_{2}},\;\gamma =\frac{a_{4}}{a_{6}},\;\kappa =\frac{K}{a_{1}},\;\delta \\ =\alpha \overset{ˇ}{C},\;\alpha =\frac{a}{a_{2}},\;\rho =\frac{a_{5}}{a_{1}},\mu =\frac{a_{7}}{a_{2}},\;\sigma =\frac{a_{8}a_{2}}{a_{1}},\;\eta =\frac{a_{9}}{a_{1}}. \end{array}$$

### 2.1 Positivity and boundedness

In this section, we showed that our solutions are positive and bounded, ensuring that the results are biologically feasible and robust.

Theorem 2.1: The solution x(τ) and y(τ) of system (Equation 4) is positive ∀ τ>0 , if x(0)>0 and y(0)>0 (67, 68).

*Proof.* Let x(0)>0 and y(0)>0 . Then, from the first equation of system (Equation 4), we write:

$\frac{dx}{d\tau }=x\left(1-x\right)-\beta x\left(\frac{y}{\gamma +y}\right)-\frac{\kappa x}{\alpha +\delta x}.$ Now define,


$$M_{1}=\underset{\tau \geq 0}{\max}\left\{\beta \left(\frac{y}{\gamma +y}\right)+\frac{\kappa }{\alpha +\delta x}\right\}\leq \;\beta +\frac{\kappa }{\alpha }.$$

So,


$$\frac{dx}{d\tau }\geq x\left(-x-M_{1}\right)=-x\left(x+M_{1}\right).$$

Now, consider


$$\frac{dz}{d\tau }=-z\left(z+M_{1}\right),\;z\left(0\right)=\;x_{0}>0.$$

This is a standard Ricatti-type inequality that ensures z(τ)>0, and by comparison theorem (69, 70)


x(τ)≥z(τ)>0 ⇒ x(τ)>0 ∀ τ>0.

Similarly, from the second equation of system (Equation 4),


$$\frac{dy}{d\tau }=\rho y\left(1-y\right)\left(\frac{x}{\mu +x}\right)-\sigma xy-\eta y.$$

Clearly, the right-hand side has the form:


$$\frac{dy}{d\tau }\geq -y\left(\sigma x+\eta \right)\geq -M_{2}y,\;\;\;\;\text{where }M_{2}=\underset{\tau \geq 0}{\max}\left(\sigma x+\eta \right).$$

So we consider,


$$\frac{dz}{d\tau }=-M_{2}z,\;z\left(0\right)=y_{0}>0\;\Rightarrow \;z\left(\tau \right)=y_{0}e^{-M_{2}\tau }>0.$$

Then,


y(τ)≥z(τ)>0 ⇒ y(τ)>0 ∀ τ>0.

Thus, x(τ)>0 and y(τ)>0 , implies that all solutions are positive.

Theorem 2.2: The solution x(τ) and y(τ) of system (Equation 4) is bounded ∀ τ≥0 (69, 70).

*Proof.* We aim to show that x(τ) and y(τ) are bounded for τ≥0 . From the first equation,


$$\begin{array}{l} \;\frac{dx}{d\tau }=x\left(1-x\right)-\beta x\left(\frac{y}{\gamma +y}\right)-\frac{\kappa x}{\alpha +\delta x}\\ \Rightarrow \;\frac{dx}{d\tau }\leq x\left(1-x\right). \end{array}$$

This is a classical logistic equation. The solution is bounded above by 1. So, by comparison theorem (70),


x(τ)≤1 if x(0)≤1, and x(τ)→1 as τ→∞.

Therefore, x(τ)≤max{1, x(0)},  so x(τ) is bounded.

Similarly, for the second equation,


$$\frac{dy}{d\tau }=\rho y\left(1-y\right)\left(\frac{x}{\mu +x}\right)-\sigma xy-\eta y.$$

We have, $\frac{dy}{d\tau }\leq \rho y\left(1-y\right)$ . Thus, y(τ)≤1 if y(0)≤1,  and y(τ)→1 as τ→∞. .

Thus, y(τ)≤max{1, y(0)},  so y(τ) is bounded. Therefore, x(τ) and y(τ) are bounded.

### 2.2 Equilibrium analysis

In this section, we find the equilibrium points and state the conditions for their existence. To evaluate these points, we set the right-hand side of model (4) to zero and solve simultaneously for x and y .


$$x\left(1-x\right)-\beta x\left(\frac{y}{\gamma +y}\right)-\frac{\kappa x}{\alpha +\delta x}=0$$

$$\rho y\left(1-y\right)\left(\frac{x}{\mu +x}\right)-\sigma xy-\eta y=0.$$

#### 2.2.1 Cancer-free equilibrium

The first steady state, referred to as the cancer-free equilibrium $E_{0}=\left(0,\;0\right)$ , occurs when both the cancer and immune cells are eradicated (71, 72).

#### 2.2.2 Coexistence equilibrium

The second steady state is the coexistence equilibrium (73, 74) obtained as follows:


(5) $$x\left(1-x\right)-\beta x\left(\frac{y}{\gamma +y}\right)-\frac{\kappa x}{\alpha +\delta x}=0\;$$

(6) $$\rho y\left(1-y\right)\left(\frac{x}{\mu +x}\right)-\sigma xy-\eta y=0.$$

We aim to express the coexistence equilibrium values x∗ and y∗, such that x∗ > 0 and y∗ > 0, with y∗ in terms of x∗. Starting with (Equation 6), we solve for y∗ in terms of x∗:


$$\begin{array}{l} \;\rho \left(1-y^{*}\right)\left(\frac{x^{*}}{\mu +x^{*}}\right)=\sigma x^{*}+\eta \\ \Rightarrow \;\left(1-y^{*}\right)=\frac{\left(\mu +x^{*}\right)\left(\sigma x^{*}+\eta \right)}{\rho x^{*}}. \end{array}$$

$$\Rightarrow \;y^{*}=1-\frac{\left(\mu +x^{*}\right)\left(\sigma x^{*}+\eta \right)}{\rho x^{*}}.$$

Now substitute this expression in (Equation 5) we have,


$$1-x^{*}-\beta \left(\frac{y^{*}}{\gamma +y^{*}}\right)=\frac{\kappa x^{*}}{\alpha +\delta x^{*}},$$

where $y^{*}=1-\frac{\left(\mu +x^{*}\right)\left(\sigma x^{*}+\eta \right)}{\rho x^{*}}.$ This yields a single nonlinear equation in terms of $x^{*}$ , which can be solved numerically to obtain the coexistence steady state.

This steady state offers insights into the potential long-term dynamics of the disease and may guide strategies for its control and management.

### 2.3 Stability analysis

We now assess the local stability of the model at the death equilibrium point by examining the eigenvalues of the Jacobian matrix evaluated at this steady state. Consider the Jacobian matrix given by:


(7) $$J\left(x,\;y\right)=\left[\begin{array}{cc} 1-2x-\frac{\beta y}{\left(\gamma +y\right)}-\frac{\kappa \alpha }{{\left(\alpha +\delta x\right)}^{2}} & -\frac{\beta \gamma x}{{\left(\gamma +y\right)}^{2}}\\ \rho y\left(1-y\right)\left(\frac{\mu }{{\left(\mu +x\right)}^{2}}\right)-\sigma y & \frac{x}{\mu +x}\left(\rho -2\rho y\right)-\sigma x-\eta \end{array}\right]$$

Theorem 2.3: The steady state $E_{0}$ is locally asymptotically stable if κ>α (75, 76).

*Proof.* Evaluating the Jacobian matrix (7) at the death steady state $E_{0}=\left(0,\;0\right),$ we have (75):


$$J\left(0,\;0\right)=\left[\begin{array}{cc} 1-\frac{\kappa }{\alpha } & 0\\ 0 & -\eta \end{array}\right].$$

Thus, the eigenvalues of J(0, 0) are given by $\lambda_{1}=1-\frac{\kappa }{\alpha }$ and $\lambda_{2}=-\eta .$ Observe that $\lambda_{2}<0$ and $\lambda_{1}<0$ if and only if κ>α. Thus, the steady state $E_{0}$ is locally asymptotically stable if κ>α (76).

Theorem 2.4: The steady state $E_{0}$ is globally asymptotically stable if κ>α and $\frac{\rho }{\mu }<\frac{\beta }{\gamma }+\sigma$ (77).

Proof. Define the following linear function by


V(x,y)=x+y.

Observe that this function V(x,y)>0 for all (x,y)≠(0, 0), V(0, 0)=0,  and radially unbounded (x+y→∞, V→∞) . Therefore, V(x, y) is positive definite and hence a Lyapunov function (77). Now,


$$\frac{dV}{d\tau }=\frac{dx}{d\tau }+\frac{dy}{d\tau }.$$

Substituting the system equations we have,


$$\begin{array}{r} \frac{dV}{d\tau }=\left[x\left(1-x\right)-\beta x\left(\frac{y}{\gamma +y}\right)-\frac{\kappa x}{\alpha +\delta x}\right]+\left[\rho y\left(1-y\right)\left(\frac{x}{\mu +x}\right)-\sigma xy-\eta y\right]\\ \leq x-\frac{\beta xy}{\gamma }-\frac{\kappa x}{\alpha }+\frac{\rho xy}{\mu }-\sigma xy-\eta y\\ =x\left(1-\frac{\kappa }{\alpha }\right)+y\left[x\left(\frac{\rho }{\mu }-\frac{\beta }{\gamma }-\sigma \right)-\eta \right]<0\text{ if }\kappa >\alpha \text{ and }\frac{\rho }{\mu }<\frac{\beta }{\gamma }+\sigma . \end{array}$$

Thus, $\frac{dV}{d\tau }<0$ if κ>α and $\frac{\rho }{\mu }<\frac{\beta }{\gamma }+\sigma .$ Hence, the equilibrium point is globally asymptotically stable (77).

## 3 Sensitivity analysis

In this section, we conduct a sensitivity analysis, an important technique for assessing how the model’s solution responds to changes in its parameters. We examine the sensitivity of tumor concentration to each parameter, evaluating it through forward sensitivity index analysis.

**Figure 1:**
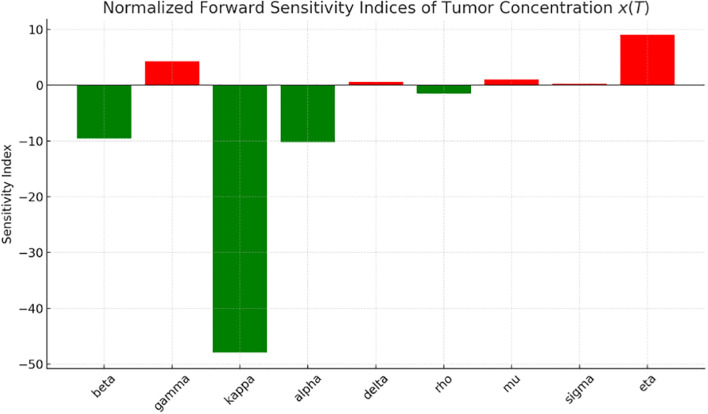
Normalized forward sensitivity index.

**Figure 1** presents the graph of the sensitivity analysis. The bar plot illustrates the sensitivity of tumor concentration x(T) to each parameter. Parameters shown in green reduce tumor concentration when increased, whereas those in red cause it to rise. This visual quickly allows identification of the parameters are most influential for control strategies. The most influential parameter in reducing tumor is κ (effect of early detection), followed by α (awareness) and (β, ρ) immune response. Enhancing awareness and immune response reduces tumor concentration, as shown in **Figure 1**. High immune decay (η) or low immune effectiveness (γ) increases tumor burden. **Table 1** provides a detailed description of the sensitivity analysis.

**Table 1:** Sensitivity index.

Parameter	Sensitivity index	Interpretation
β	−9.57	Tumor decreases strongly with higher immune effectiveness.
γ	+4.28	Tumor increases with more immune saturation (less efficient killing).
κ	−47.92	Tumor decreases sharply with stronger early detection/removal.
α	−10.18	Tumor decreases as awareness (early detection factor) improves.
δ	+0.57	Tumor increases slightly with higher nonlinearity in early detection.
ρ	−1.49	Tumor decreases moderately with more immune activation.
μ	+1.00	Tumor increases as immune activation saturates earlier.
σ	+0.25	Tumor increases slightly with higher immune cell death from tumor.
η	+8.97	Tumor increases significantly with higher immune cell decay.

## 4 Numerical simulations

In this section, we use the parameter values from **Table 2** to present the numerical simulations of model (2) showing the impact of early detection on tumor growth, using MATLAB version 2017b to draw **Figures 1**–**12** (25, 78, 79).

**Table 2:** Parameter values.

Parameter	Optimized value	Unit	Reference/source
β	0.5	$time^{-1}$	Kaur et al. (19), Mohammad and Arafa (20), Ryser et al. (25), Siegel et al. (36):
γ	0.1	Dimensionless	Kaur et al. (19)
κ	3.0	$time^{-1}$	Arafa and Elnaggare (42)
α	0.65	Dimensionless	Optimized, WHO reports, Arafa and Elnaggare (42), Ryser et al. (25), Siegel et al. (36)
δ	1.0	Dimensionless	Assumed
ρ	1.0	$time^{-1}$	Singh et al. (43), Kaur et al. (19)
μ	0.1	Dimensionless	Kaur et al. (19)
σ	0.3	$cell^{-1}time^{-1}$	Singh et al. (43)
η	0.1	$time^{-1}$	Mohammad and Arafa (20)
x(0)	0.35	Dimensionless	Optimized (within 0.1–0.5 range)
y(0)	0.08	Dimensionless	Optimized (within 0.01–0.2 range)
τ	0–50	time	Assumed

**Figure 2:**
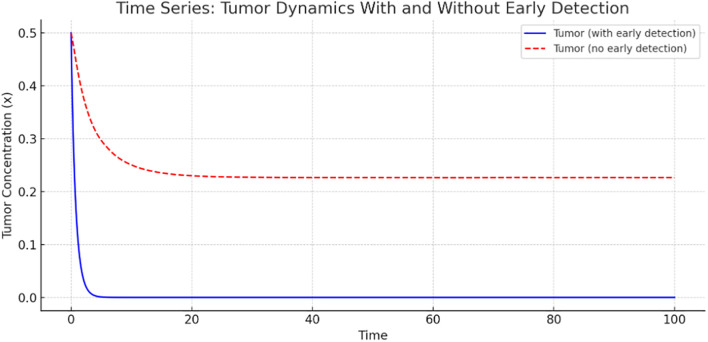
Tumor dynamics with and without early detection.

**Figure 3:**
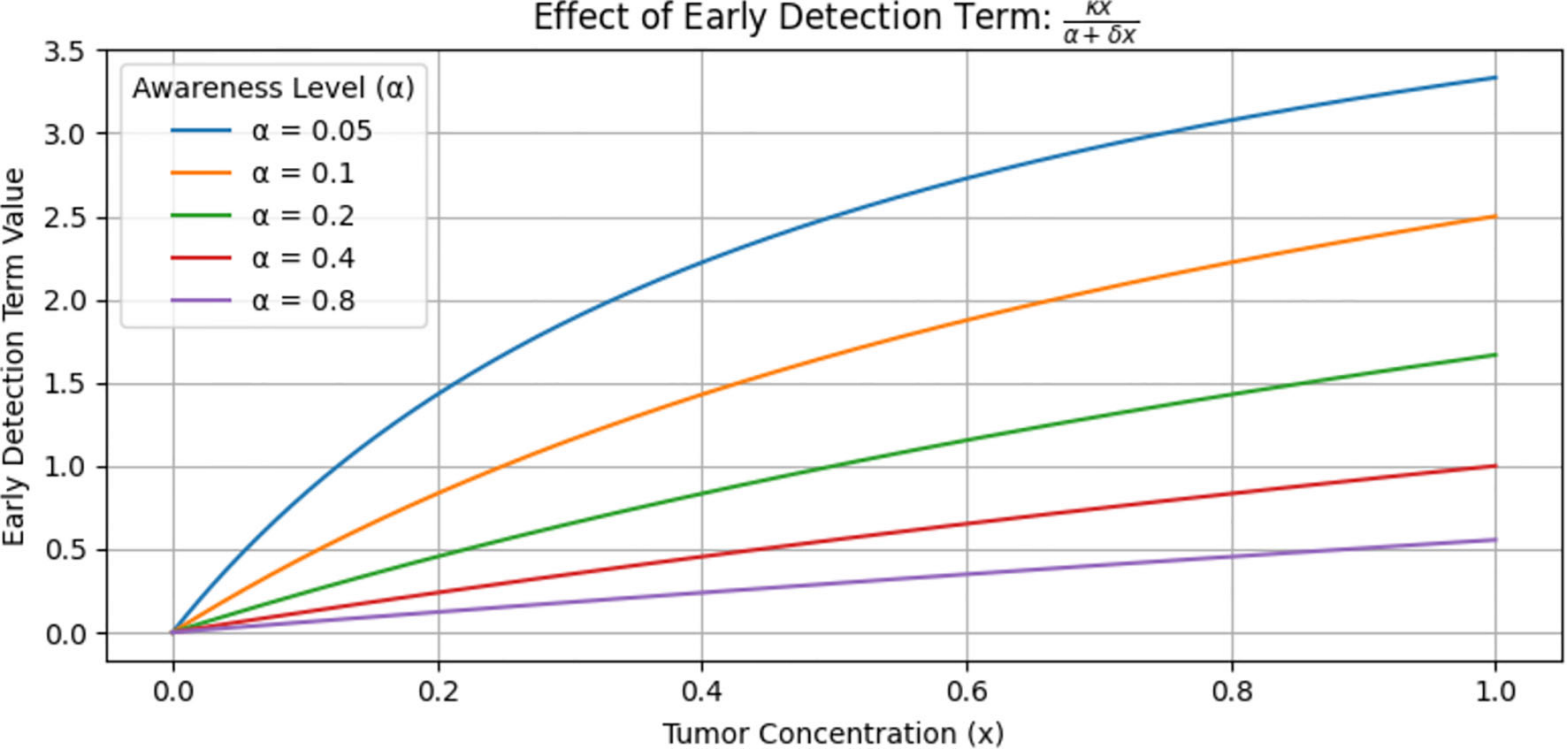
Effect of early detection on tumor size.

**Figure 4:**
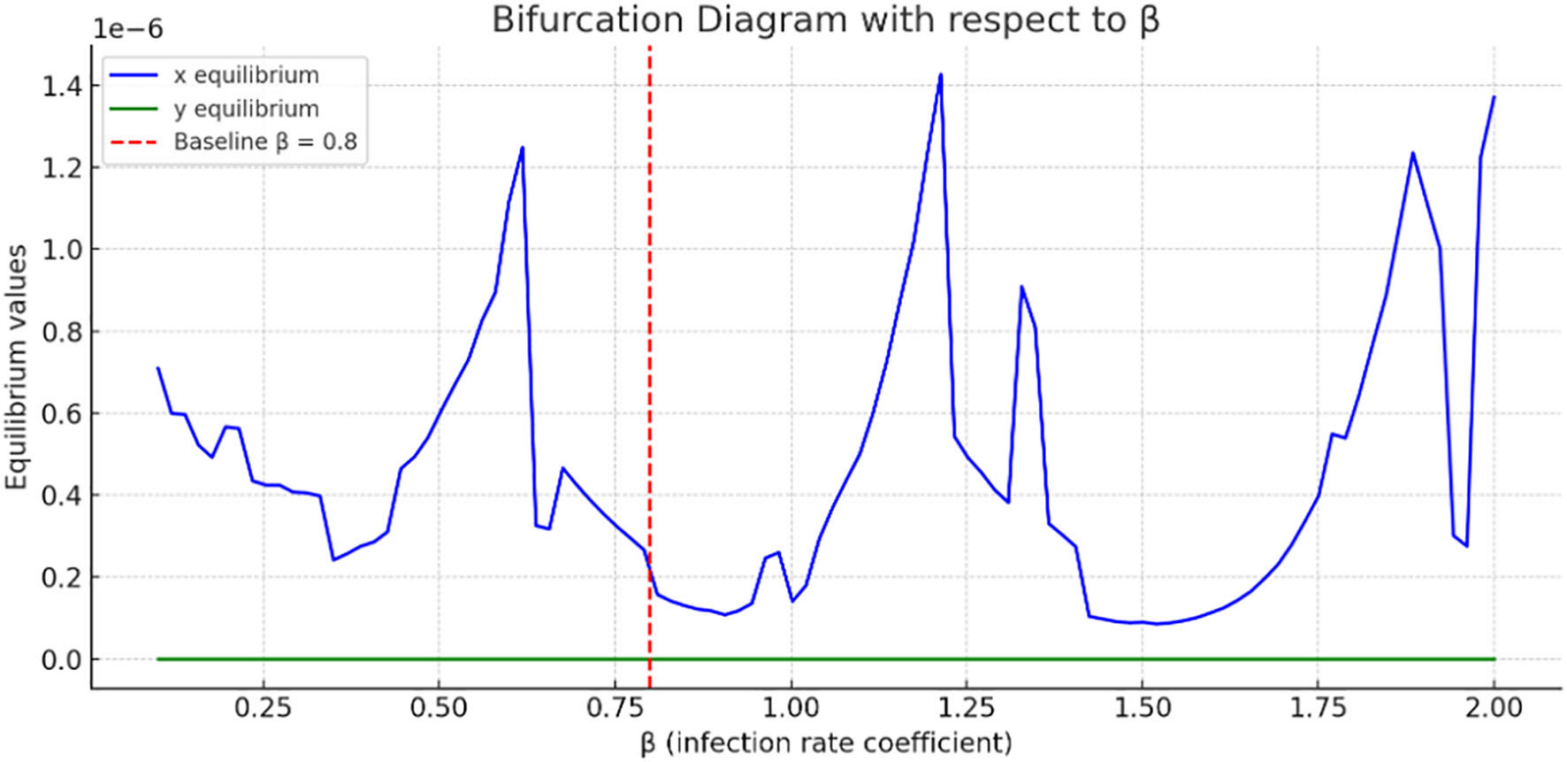
Bifurcation analysis with respect to β.

**Figure 5:**
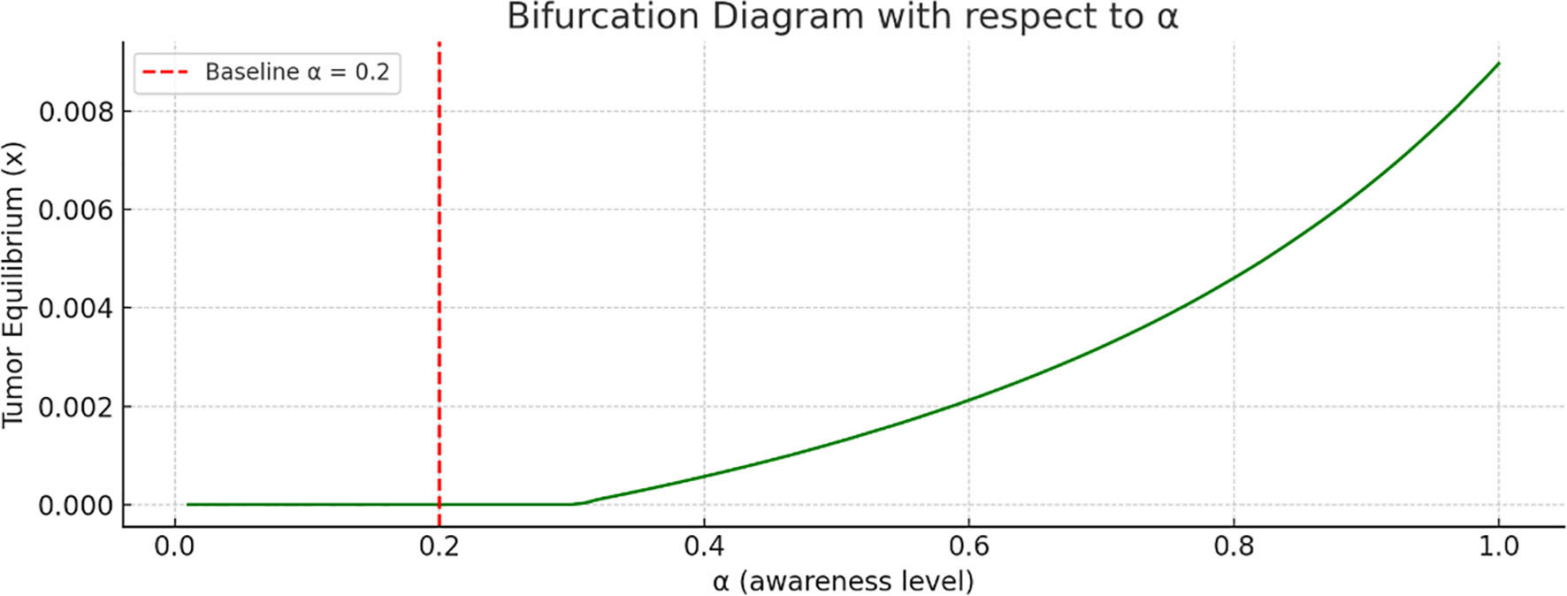
Bifurcation analysis with respect to α.

**Figure 6:**
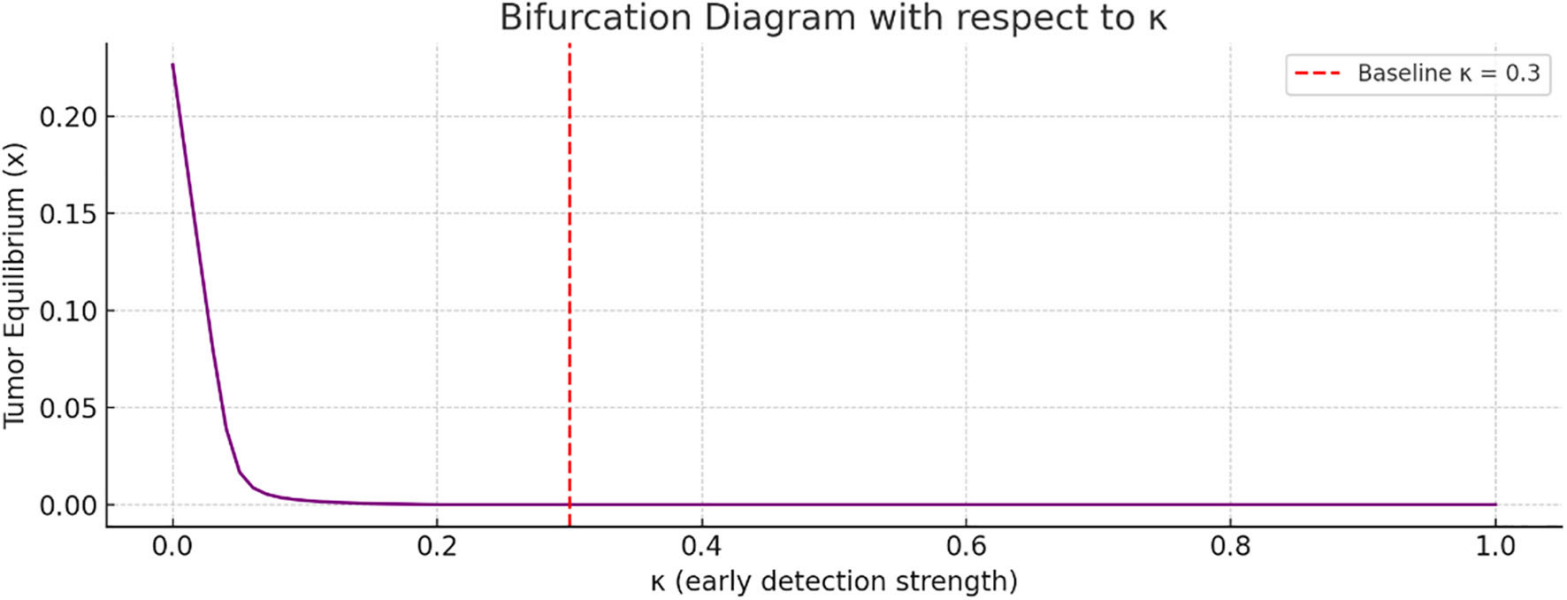
Bifurcation analysis with respect to κ.

**Figure 7:**
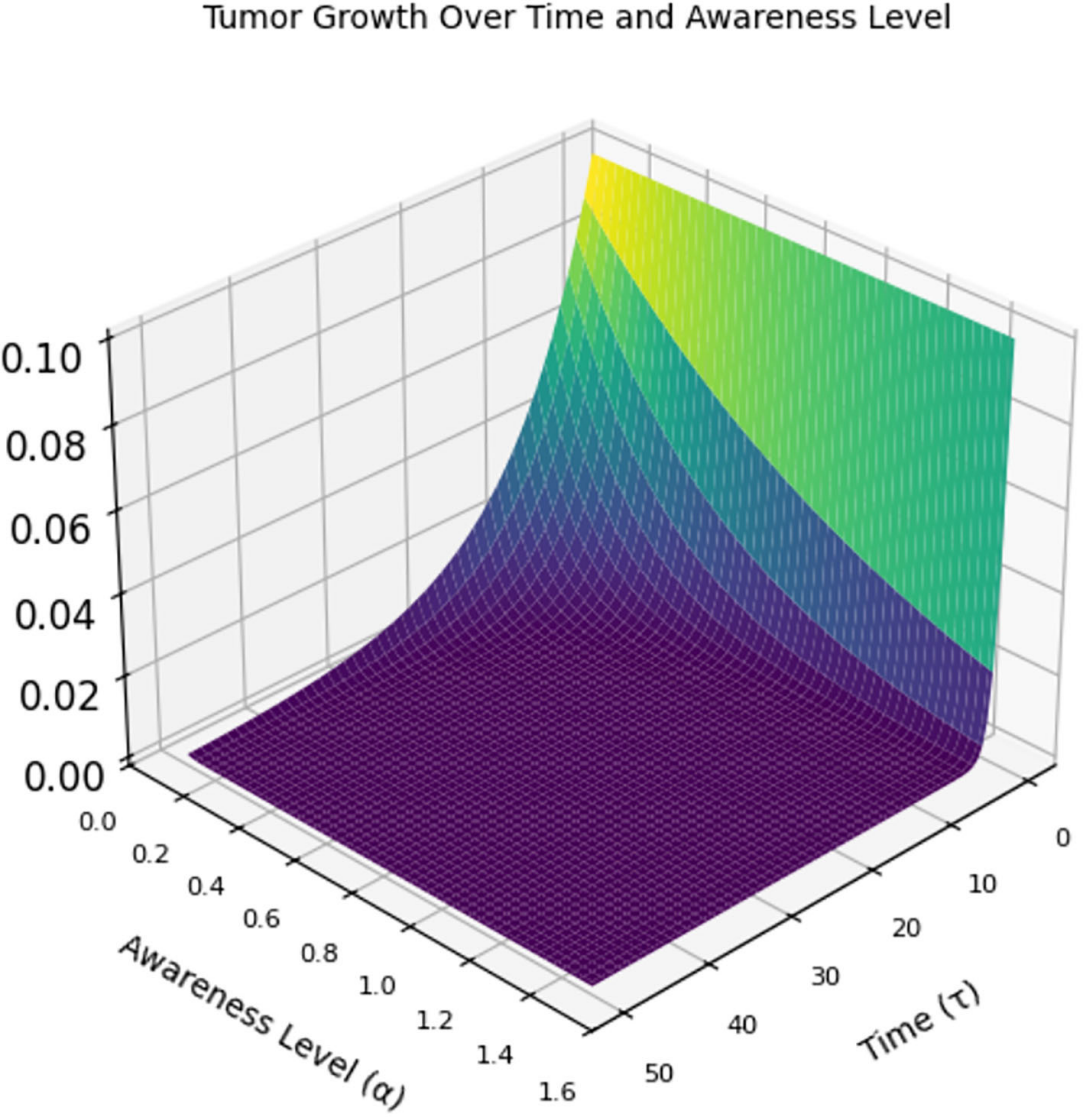
3D graph of tumor growth.

**Figure 8:**
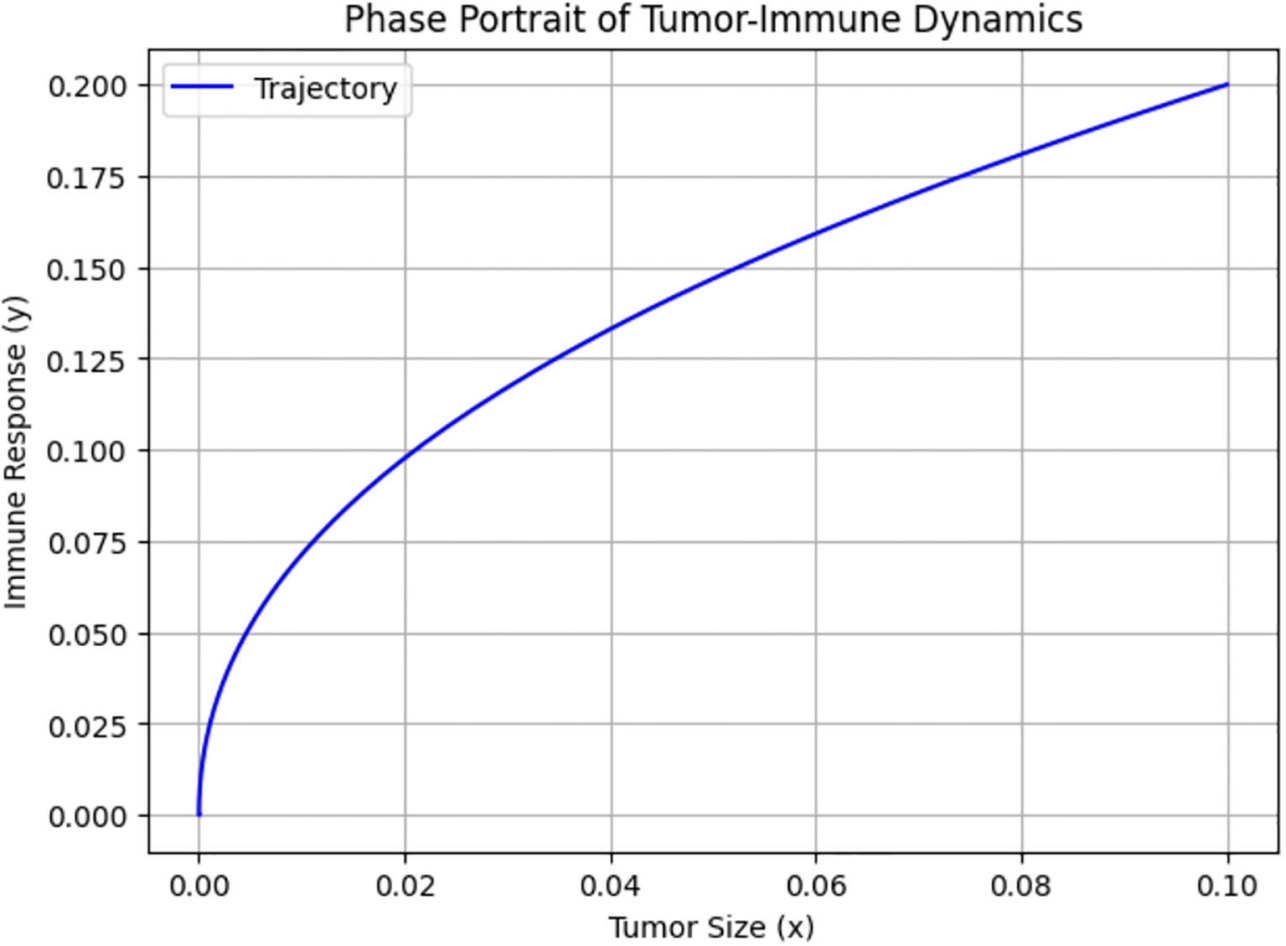
Phase portrait of tumor-immune dynamics.

**Figure 9:**
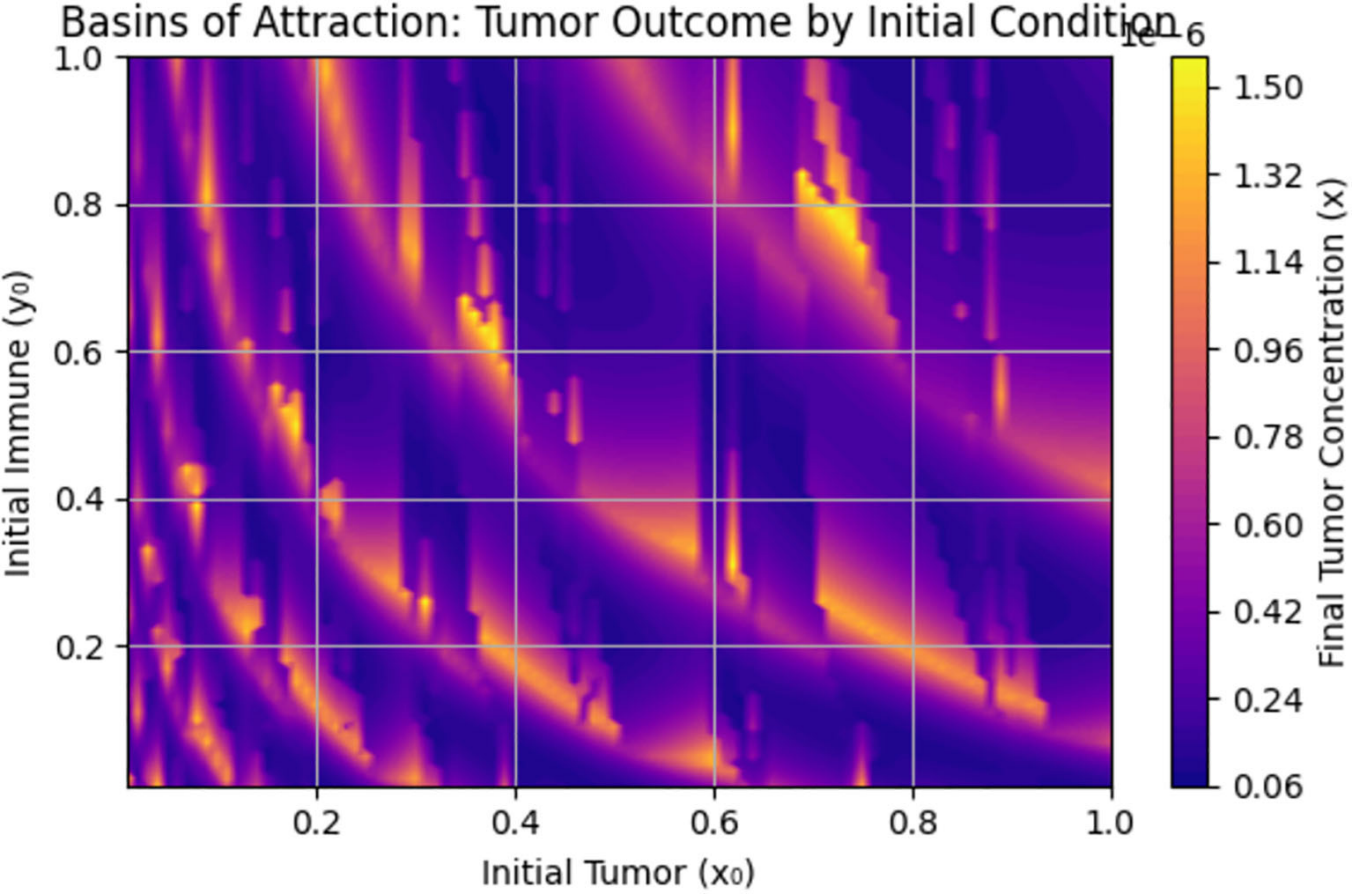
Basin of attraction.

**Figure 10:**
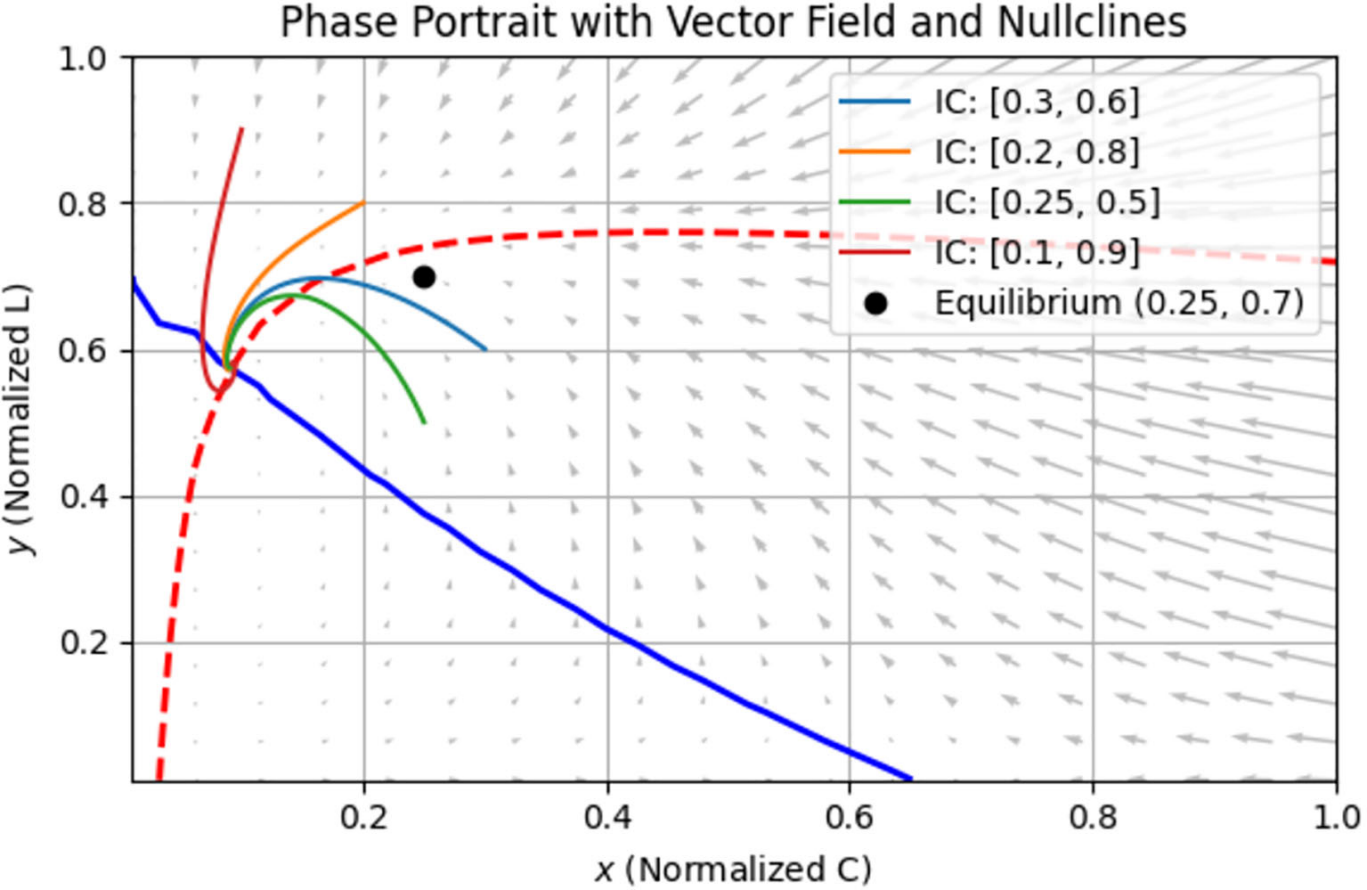
Phase portrait with vector field and nullclines.

**Figure 11:**
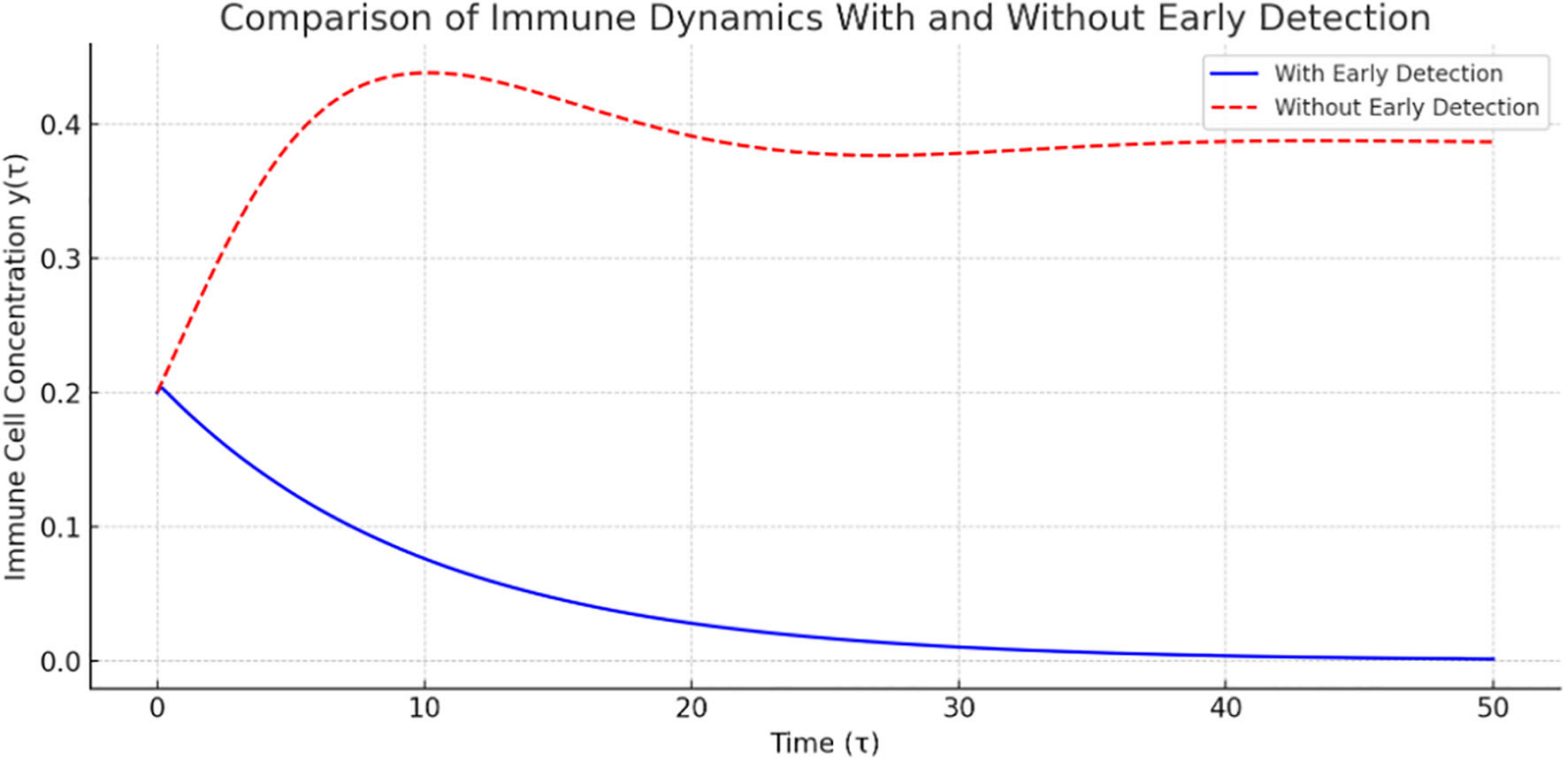
Immune dynamics with and without early detection.

**Figure 12:**
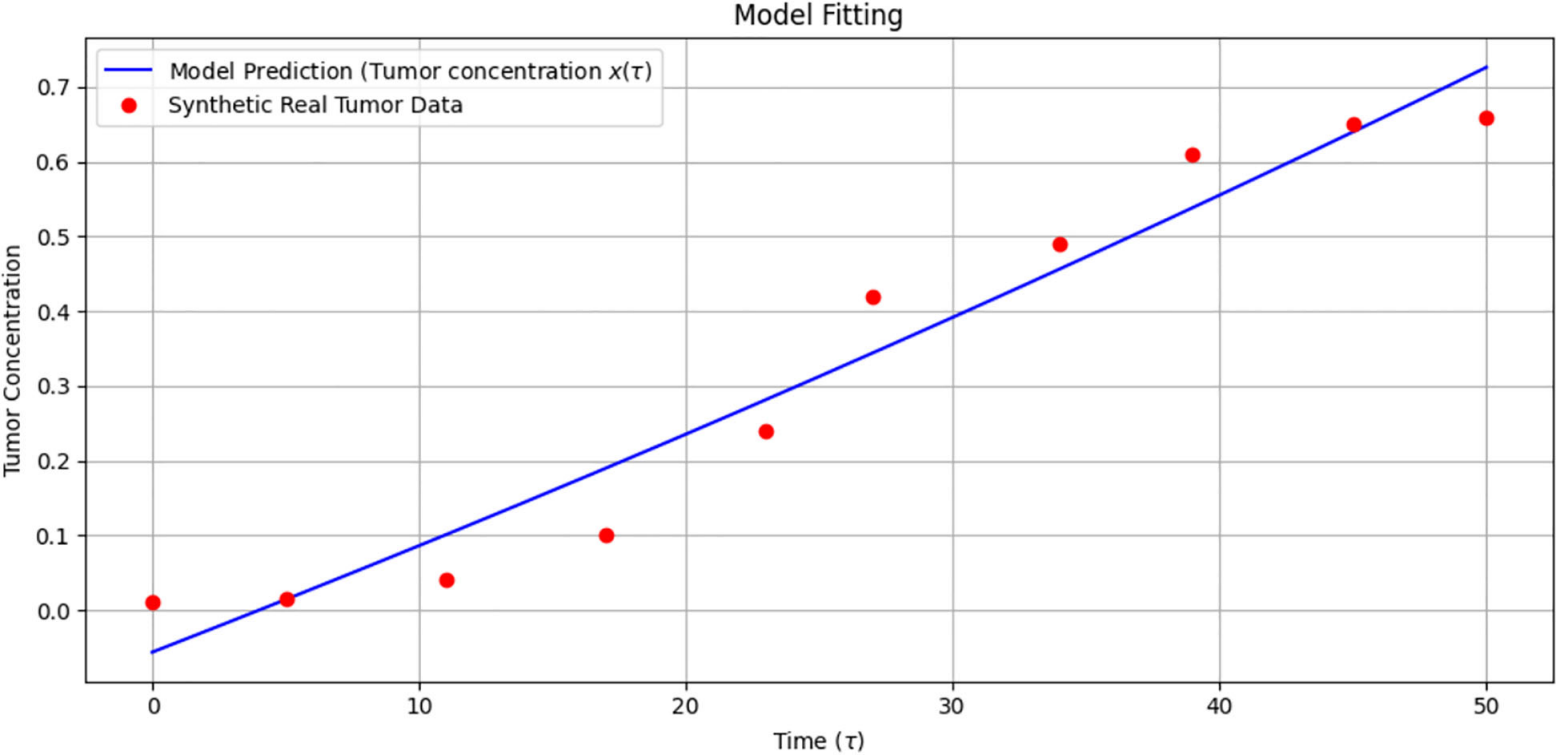
Model fitting of tumor concentration over time.

**Figure 2** presents a time-series comparison of tumor concentration with and without early detection. The tumor shrinks rapidly to nearly zero, indicating that early detection is highly effective in controlling tumor growth. However, without early detection, tumor concentration stabilizes at a higher level, indicating persistent tumor presence. This graph clearly illustrates the significant role of early detection in reducing tumor burden.

**Figure 3** shows the value of the early detection term, which is a function of tumor concentration at various levels. This graph does not show the tumor level but rather the effectiveness of the early detection term. The blue curve (α = 0.05) lies higher, meaning that at every level of x , the early detection term is stronger and removes more tumor. In contrast, the purple curve (α = 0.8) lies lower, meaning that at every level of x , the early detection term is weaker and removes less tumor. When small α (i.e., high awareness) implies a stronger early detection term, which leads to greater tumor reduction. Conversely, a large α (i.e., low awareness) implies a weaker detection term, resulting in less effective tumor reduction. In general, stronger early detection leads to greater tumor reduction.

**Figure 4** present a bifurcation diagram illustrating how the equilibrium values of the system’s variables change as the parameter β (tumor–immune interaction rate) varies. The blue curve fluctuates dramatically, suggesting complex tumor dynamics as β increases. Multiple bifurcation points may occur, where small changes in β lead to large shifts in tumor’s steady state. Tumor concentration values are on the order of $10^{-6}$ , indicating very small steady-state tumor populations**;** however, they are not identically zero, showing persistence of tumor even under high immune killing.

The green curve, representing immune cells, is almost flat and near zero, indicating minimal change in immune cell equilibrium with varying β . This might suggest immune suppression or exhaustion in the model. Sharp oscillations in the curve indicate nonlinear, and possibly chaotic, tumor responses to changes in β . The bifurcation analysis used the cancer-free equilibrium $E_{0}=\left(0,\;0\right)$ , and the observed transition illustrates a forward (supercritical) bifurcation with respect to β .

Bifurcation analysis with respect to the awareness level α is shown in **Figure 5**. As the awareness parameter increases, the tumor equilibrium decreases. This indicates that a higher level of awareness, more effective campaigns, or earlier screening significantly reduce tumor size. There is no sharp bifurcation point; however, there is a continuous decline in tumor equilibrium.

**Figure 6** referred to bifurcation analysis with respect to the early detection strength κ . As κ increases, the tumor equilibrium value decreases. This implies that stronger early detection significantly suppresses tumor growth. The transition appears smooth, without any abrupt bifurcation point.

**Figure 7** is a 3D graph of time (x-axis), awareness level (y-axis), and tumor size (z-axis). At low awareness levels (left side of the y-axis), the tumor starts at a certain size and grows rapidly over time (higher z-values). When awareness increases (moving right on the y-axis), the tumor shrinks more quickly. This implies that at high awareness levels, the tumor is detected early and controlled, leading to the tumor size dropping to almost zero early and remaining suppressed throughout the time span.

**Figure 8** shows the phase portrait of Tumor–Immune Dynamics, where the x- and y-axes represent the concentrations of tumor cells and immune cells actively engaged in tumor suppression, respectively. The trajectory illustrates how the state of the system evolves over time in the phase space defined by tumor size and immune response. It starts with a low tumor burden and low immune activity, and as time progresses, the system moves along the path shown.

Biologically, this indicates that both tumor cells and immune response start at low levels, representing a situation immediately after tumor initiation, when the tumor is small and not yet strongly detected by the immune system. The progression along the curve implies that as tumor size increases slightly, the immune system begins to activate. The upward trajectory suggests that the immune system is responding proportionally to the tumor presence. The curve indicates a positive, nonlinear relationship between tumor size and immune response. This suggests that the immune system becomes increasingly responsive as tumor cells proliferate. This aligns with known biological phenomena, in which the immune system often remains inactive until the tumor burden exceeds a certain threshold that triggers recognition and response. The curve gradually flattens, indicating that even as tumor size grows, the immune response increases more slowly. This indicates immune exhaustion or checkpoint inhibition effects. It also represents suboptimal immune activation, where increasing tumor size is not met with a proportionate immune escalation.

The basin of attraction in **Figure 9** shows how the final tumor concentration depends on the initial conditions of the system—specifically the initial tumor cell population x(0) and initial immune cell population (0) . When y(0) is moderately high (e.g., >0.5) and x(0) is not too high, the tumor is often reduced to low final levels (dark purple). This indicates that immune cells successfully suppress tumor growth. Light yellow zones indicate initial conditions where the tumor persists or grows, possibly due to low initial immune presence, a large initial tumor load overwhelming the immune system, or insufficient early detection (κ, α) .

**Figure 9** underscores the importance of early immune intervention and tumor detection. There are immune presence thresholds below which the tumor cannot be controlled. This supports the value of early detection campaigns and strategies to boost immune function in breast cancer treatment.

**Figure 10** gives the phase portrait with vector fields, nullclines, and trajectories for system (3). The vector field (gray arrows) shows the direction of movement or dynamics of the system (small arrows). It illustrates how the state of the system changes over time. For example, at a point (x,y) , the arrow indicates how tumor and immune levels change next.

For the nullclines, we can observe the tumor nullcline, where tumor size does not change (blue line), the immune nullcline (red dashed line), and their intersection (black dot), which gives the coexistence steady state. The coexistence steady state, as shown in the graph, is (0.25, 0.70). If the system reaches this point, it will remain there. This tells us that the tumor stabilizes at 25% maximum size, while the immune response stabilizes at 70%. Each line represents a simulation from different initial conditions (IC) described in **Table 3**.

**Table 3:** Simulation from different initial conditions.

Color	Initial condition (x,y)	Interpretation
Blue	[0.3, 0.6]	Higher tumor start, moderate immune response
Orange	[0.2, 0.8]	Moderate tumor, strong immune
Green	[0.25, 0.5]	Lower immune start
Red	[0.1, 0.9]	Low tumor, high immune

Observe that all trajectories converge toward the equilibrium point, confirming local stability. The direction of flow confirms that the system naturally regulates tumor growth, depending on the strength of the immune response. In conclusion, this phase portrait demonstrates stable tumor–immune dynamics: the immune system can contain the tumor at moderate levels, depending on initial conditions, and the system is locally stable around the equilibrium point (0.25, 0.7), validating our stability analysis.

**Figure 11** compares the evolution of immune cell concentrations over time under two scenarios—with and without early detection. Early detection reduces tumor burden, which in turn lowers immune stimulation and causes a decrease in immune cell levels. In contrast, the absence of early detection results in a more active tumor presence, which sustains higher immune response levels over time. This figure clearly demonstrates the impact of early detection in reducing immune activation demand, thereby supporting the effectiveness of early cancer diagnosis in controlling immune–tumor dynamics.

**Figure 12** demonstrates the model fitting of tumor concentration over time. The blue line represents the mathematical model prediction x(τ) , and the red dots represent synthetic real tumor data. The model provides a good fit, effectively capturing tumor growth dynamics over the observed time period.

Due to the lack of access to patient-specific tumor concentration data, the data used for model fitting are synthetic, designed to emulate realistic growth trends. This approach is supported in the literature as a valid method for validating mathematical oncology models (16, 25).

## 5 Discussion and conclusion

We consider a mathematical model of breast cancer with cytotoxic lymphocytes, incorporating the effect of early detection in treating the disease. The model was nondimensionalized to simplify analysis by reducing the number of parameters involved. Two steady states were obtained: cancer-free state and a coexistence states. Local and global stability analyses were conducted using a suitable Lyapunov function, and conditions for stability was stated. A sensitivity analysis was conducted to determine how sensitive the solution of the model is to changes in parameters.

We explored the sensitivity of tumor concentration to each parameter using the forward sensitivity index analysis. **Figure 1** shows the sensitivity analysis graph, where the most influential parameter in reducing tumor is κ (effect of early detection), followed by α (awareness), and β, ρ (immune response). Improving awareness and immune response helps reduce tumor concentration, as observed in **Figure 1**, while high immune decay (η) or low immune effectiveness (γ) worsens tumor burden. A comparison of tumor concentration with and without early detection shows that the tumor shrinks rapidly to nearly zero, indicating that the early detection mechanism is highly effective at controlling tumor growth. However, tumor concentration stabilizes at a higher level, indicating its persistent presence due to the lack of early detection intervention. These results clearly demonstrate the significant role of early detection in reducing tumor burden.

More so, bifurcation diagrams were presented with regards to β (immune response), κ (early detection parameter), and α (awareness campaign). As the awareness and early detection parameters increase, the tumor equilibrium decreases, indicating that higher levels of awareness, more effective campaigns, or earlier screening significantly reduce tumor size. **Figure 9** shows the basin of attraction, emphasizing the importance of early immune intervention and tumor detection. There are thresholds of immune presence below which the tumor cannot be controlled. This supports the value of early detection campaigns and immune function enhancement as clinical strategies in breast cancer treatment.

In conclusion, despite the threat, severity, and deadly nature of breast cancer, early detection and awareness campaigns are key to curing or improving patients’ lives, as indicated by the mathematical model and supported by numerical simulations in this study. Thus, relevant authorities should emphasize the need to strengthen early detection and awareness campaigns among the populace to curb the menace posed by this deadly disease.

## 6 Limitations

We believe that the current model provides valuable insights into the interplay between tumor dynamics and immune response, particularly under the influence of awareness-driven early detection; however, it has some limitations:

- The immune response is modeled as a single population of immune cells. In reality, the immune system comprises various components—such as helper T cells, cytotoxic T cells, and regulatory T cells—that interact in complex ways not captured in this study.
- The model does not account for *time delays* in immune activation, tumor recognition, or treatment response. Such delays can significantly affect system dynamics and stability.
- The model does not incorporate control or treatment optimization. Although early detection is considered, optimal intervention strategies—such as optimal control, drug therapy schedules, or awareness campaign intensities—are not explored in this study.
- The model does not capture memory effects using the concept of fractional calculus.
- Another key limitation is the lack of real-world patient data integration. All parameter values in this study are based on literature or reasonable assumptions. We acknowledge that empirical validation is essential for enhancing the predictive and practical relevance of the model.
- Due to the complex nonlinear structure of the model and the algebraic complexity of expressions at the coexistence equilibrium, it was not analytically tractable to determine stability using classical mathematical techniques such as linearization or Lyapunov analysis.

## 7 Future research directions

To address these limitations and enhance the model, future research could explore the following avenues:

- Incorporate multiple immune compartments: Extend the model to include multiple immune cell types and cytokine interactions, capturing more detailed tumor-immune dynamics.
- Introduce time delay differential equations: Investigate the impact of immune response delays and diagnosis/treatment lag on tumor control and system stability.
- Add spatial dimensions via PDEs or agent-based models: Develop spatially explicit models or hybrid approaches that combine ODEs with agent-based modeling (ABM) to simulate spatial structure and tumor–immune heterogeneity.
- Explore optimal control strategies: Introduce control functions for early detection efforts, immunotherapy dosing, awareness campaigns, or a combination of these to identify cost-effective strategies for tumor suppression using Pontryagin’s Maximum Principle or numerical optimal control methods.
- Compare with clinical data for specific cancer types: Calibrate and validate the model using real patient data from breast cancer or other tumor types to improve relevance and potential for clinical application.
- Incorporate real patient datasets: As part of future research, extend the model to incorporate real patient datasets. This will enable parameter fitting, model refinement, and more robust empirical validation, enhancing applicability in clinical and public health decision-making.

## Data Availability

The original contributions presented in the study are included in the article/supplementary material. Further inquiries can be directed to the corresponding author.

## Appendix

**1. Nondimensionalization**

Consider the dimensionalized model equations below:

$\frac{dC\left(t\right)}{dt}=a_{1}C\left(t\right)\left(1-\frac{C\left(t\right)}{a_{2}}\right)-a_{3}C\left(t\right)\left(\frac{L\left(t\right)}{a_{4}+L\left(t\right)}\right)-\frac{KC\left(t\right)}{a+\alpha \tilde{C}C\left(t\right)},$ $\frac{dL\left(t\right)}{dt}=a_{5}L\left(t\right)\left(1-\frac{L\left(t\right)}{a_{6}}\right)\left(\frac{C\left(t\right)}{a_{7}+C\left(t\right)}\right)-a_{8}C\left(t\right)L\left(t\right)-a_{9}L\left(t\right).$ To nondimensionalize this model, we make the following change of variables as follows:

Let $x=\frac{C}{a_{2}},\;y=\frac{L}{a_{6}},$ and $\tau =a_{1}t.$ Then, $C=a_{2}x,\;L=a_{6}y,$ also $\frac{dC}{dt}=a_{2}.a_{1}\frac{dx}{d\text{τ}}$ and $\frac{dC}{dt}=a_{6}.a_{1}\frac{dy}{d\text{τ}}.$ Substituting in the first and second equation of the model we have,

$a_{2}.a_{1}\frac{dx}{d\text{τ}}=a_{2}.a_{1}x\left(1-\frac{a_{2}x}{a_{2}}\right)-a_{3}a_{2}x\left(\frac{a_{6}y}{a_{4}+a_{6}y}\right)-\left(\frac{Ka_{2}x}{a+\alpha \tilde{C}a_{2}x}\right),$ $a_{6}.a_{1}\frac{dy}{d\text{τ}}=a_{5}.a_{6}y\left(1-\frac{a_{6}y}{a_{6}}\right)\left(\frac{a_{2}x}{a_{7}+a_{2}x}\right)-a_{8}a_{2}x.a_{6}y-a_{9}a_{6}y.$ This implies that,

$\frac{dx}{d\text{τ}}=x\left(1-x\right)-\text{β}x\left(\frac{y}{\gamma +y}\right)-\left(\frac{\kappa x}{a+\delta x}\right),$ $\frac{dy}{d\text{τ}}=\rho y\left(1-y\right)\left(\frac{x}{\mu +x}\right)-\sigma xy-\eta y,$ where $\beta =\frac{a_{3}a_{6}}{a_{1}a_{2}},\;\gamma =\frac{a_{4}}{a_{6}},\;\kappa =\frac{K}{a_{1}},\;\delta =\alpha \overset{ˇ}{C},\;\alpha =\frac{a}{a_{2}},\;\rho =\frac{a_{5}}{a_{1}},\mu =\frac{a_{7}}{a_{2}},\;\sigma =\frac{a_{8}a_{2}}{a_{1}},$ and $\eta =\frac{a_{9}}{a_{1}}.$ Thus, the nondimensionalized model is given by

$\frac{dx}{d\text{τ}}=x\left(1-x\right)-\text{β}x\left(\frac{y}{\gamma +y}\right)-\frac{\kappa x}{a+\delta x},$ $\frac{dy}{d\text{τ}}=\rho y\left(1-y\right)\left(\frac{x}{\mu +x}\right)-\sigma xy-\eta y.$ **2. Theorem 2.4:** The steady state $E_{0}$ is globally asymptotically stable if κ>α and $\frac{\rho }{\mu }<\frac{\beta }{\gamma }+\sigma$ [72 & 81].

Proof. Define the following linear function by

V(x,y)=x+y. Observe that this function V(x,y)>0 for all (x,y)≠(0, 0), V(0, 0)=0,  and radially unbounded … Therefore, V(x, y) is positive definite and hence a Lyapunov function. Now,

$\frac{dV}{d\tau }=\frac{dx}{d\tau }+\frac{dy}{d\tau }.$ Substituting the system equations we have,

$\frac{dV}{d\tau }=\left[x\left(1-x\right)-\beta x\left(\frac{y}{\gamma +y}\right)-\frac{\kappa x}{\alpha +\delta x}\right]+\left[\rho y\left(1-y\right)\left(\frac{x}{\mu +x}\right)-\sigma xy-\eta y\right].$ Observe that,

1. x(1−x)≤x. .

2. $\beta x\left(\frac{y}{\gamma +y}\right)\geq \frac{\beta xy}{\gamma +y}\geq \frac{\beta xy}{\gamma }\;\Rightarrow -\;\beta x\left(\frac{y}{\gamma +y}\right)\leq -\frac{\beta xy}{\gamma }.$ 3. $\frac{\kappa x}{\alpha +\delta x}\geq \frac{\kappa x}{\alpha }\;\Rightarrow \;-\frac{\kappa x}{\alpha +\delta x}\leq -\frac{\kappa x}{\alpha }.$ 4. $\rho y\left(1-y\right)\left(\frac{x}{\mu +x}\right)\leq \frac{\rho xy}{\mu }$ .

Adopting the inequalities in serial 1 to 4, we have

$\frac{dV}{d\tau }=\left[x\left(1-x\right)-\beta x\left(\frac{y}{\gamma +y}\right)-\frac{\kappa x}{\alpha +\delta x}\right]+\left[\rho y\left(1-y\right)\left(\frac{x}{\mu +x}\right)-\sigma xy-\eta y\right]$ $\leq x-\frac{\beta xy}{\gamma }-\frac{\kappa x}{\alpha }+\frac{\rho xy}{\mu }-\sigma xy-\eta y$ $\;\;=x\left(1-\frac{\kappa }{\alpha }\right)+y\left[x\left(\frac{\rho }{\mu }-\frac{\beta }{\gamma }-\sigma \right)-\eta \right]<0$ if κ>α and $\frac{\rho }{\mu }<\frac{\beta }{\gamma }+\sigma .$ Thus, $\frac{dV}{d\tau }<0$ if κ>α and $\frac{\rho }{\mu }<\frac{\beta }{\gamma }+\sigma .$ .

## References

[1] OPhir N Miriam E Bunimovich-Mendrazitsky S. Analysis of a breast cancer mathematical model by a new method to find an optimal protocol for HER2-positive cancer. Biosystems. (2020) 197:104191. doi: 10.1016/j.biosystems.2020.104191, PMID: 32791173

[2] Hassani H MaChado JT Avazzadeh Z Safari E Mehrabi S. Optimal solution of the fractional order breast cancer competition model. Sci Rep. (2021) 11:15622. doi: 10.1038/s41598-021-94875-1, PMID: 34341390 PMC8329307

[3] Hanahan D Weinberg RA. Hallmarks of cancer: the next generation. Cell. (2011) 144:646–74. doi: 10.1016/j.cell.2011.02.013, PMID: 21376230

[4] World Health Organization. Breast cancer: Prevention and control. Geneva: World Health Organization (2021). Available online at: https://www.who.int/news-room/fact-sheets/detail/breast-cancer (Accessed July 17, 2025).

[5] Batlle E Clevers H. Cancer stem cells revisited. Nat Med. (2017) 23:1124–34. doi: 10.1038/nm.4409, PMID: 28985214

[6] Harkness EF Astley SM Evans DG Boggis CRM. Risk-based breast cancer screening: Current and future perspectives. Int J Women’s Health. (2020) 12:1023–35. doi: 10.1016/j.bpobgyn.2019.11.005, PMID: 31848103

[7] Norton L Simon R. Tumor size, sensitivity to therapy, and design of treatment schedules. Cancer Treat Rep. (1977) 61:1307–17., PMID: 589597

[8] Pisano C Losa M Nadin A Cavalletti E Bellucci A Mongelli N. Antitumor activity and neurotoxicity of novel epothilone analogs. J Med Chem. (2005) 48:8055–61. doi: 10.1021/jm050519k

[9] Lee SJ Zelen M. Modelling the early detection of breast cancer. Ann Oncol. (2003) 14:1199–202. doi: 10.1093/annonc/mdg323, PMID: 12881377

[10] Corcoran RB Chabner BA. Application of cell-free DNA analysis to cancer treatment. New Engl J Med. (2018) 379:1754–65. doi: 10.1056/NEJMra1706174, PMID: 30380390

[11] Saad FT Hincal E. An optimal control approach for the interaction of immune checkpoints, immune system, and BCG in the treatment of superficial bladder cancer. Eur Phys J Plus. (2018) 133:241. doi: 10.1140/epjp/i2018-12092-0

[12] Schreiber RD Old LJ Smyth MJ. Cancer immunoediting: Integrating immunity’s roles in cancer suppression and promotion. Science. (2011) 331:1565–70. doi: 10.1126/science.1203486, PMID: 21436444

[13] Muenst S Läubli H Soysal SD Zippelius A Tzankov A. The immune system and cancer evasion strategies: Therapeutic concepts. J Internal Med. (2016) 279:541–62. doi: 10.1111/joim.12470, PMID: 26748421

[14] Saad FT Hincal E Kaymakamzade B. Dynamics of immune checkpoints, immune system, and BCG in the treatment of superficial bladder cancer. Comput Math Methods Med. (2017), 3573082. doi: 10.1155/2017/3573082, PMID: 29312460 PMC5684605

[15] Idrees M Alnahdi AS Jeelani MB. Mathematical modeling of breast cancer based on the caputo–fabrizio fractal-fractional derivative. Fractal Fractional. (2023) 7:805. doi: 10.3390/fractalfract7110805

[16] Altrock PM Liu LL Michor F. The mathematics of cancer: integrating quantitative models. Nat Rev Cancer. (2015) 15:730–45. doi: 10.1038/nrc4029, PMID: 26597528

[17] Idrees M Sohail A. Bio-algorithms for the modeling and simulation of cancer cells and the immune response. Bio-Algorithms Med-Syst. (2021) 17:55–63. doi: 10.1515/bams-2020-0054

[18] Qureshi S Yusuf A Aziz S. On the use of Mohand integral transform for solving fractional-order classical Caputo differential equations. J Appl Math Comput Mech. (2020) 19:99–109. doi: 10.17512/jamcm.2020.3.08

[19] Kaur M Kumar S Saini DK. Mathematical modeling and analysis of the immune response to breast cancer under immunotherapy. Chaos Solitons Fractals. (2022) 160:112273. doi: 10.1016/j.chaos.2022.112273

[20] Mohammad SA Arafa AA. A mathematical model for the treatment of breast cancer using targeted therapy and immune cells. Alexandria Eng J. (2021) 60:1083–95. doi: 10.1016/j.aej.2020.09.034

[21] Anderson AR Quaranta V. Integrative mathematical oncology. Nat Rev Cancer. (2008) 8:227–34. doi: 10.1038/nrc2329, PMID: 18273038

[22] Bellomo N Li N Maini PK. On the foundations of cancer modelling. Selected topics, speculations, and perspectives. Math Models Methods Appl Sci. (2008) 18:593–646. doi: 10.1142/S0218202508002796

[23] Eftimie R Bramson JL Earn DJ. Interactions between the immune system and cancer: a brief review of non-spatial mathematical models. Bull Math Biol. (2011) 73:2–32. doi: 10.1007/s11538-010-9526-3, PMID: 20225137

[24] Yankeelov TE Atuegwu N Hormuth D Weis JA Barnes SL Miga MI. Clinically relevant modeling of tumor growth and treatment response. Sci Trans Med. (2013) 5:187ps9–9. doi: 10.1126/scitranslmed.3005686, PMID: 23720579 PMC3938952

[25] Ryser MD Lange JM Inoue LYT Kurian AW. Integrating biomarkers and staging in early-stage breast cancer: A mathematical modeling approach. PloS Med. (2019) 16:e1002873. doi: 10.1371/journal.pmed.1002873, PMID: 31504034 PMC6736244

[26] lharrar X Barbolosi D Ciccolini J Meille C Faivre C Lacarelle B. A phase Ia/Ib clinical trial of metronomic chemotherapy based on a mathematical model of oral vinorelbine in metastatic non-small cell lung cancer and Malignant pleural mesothelioma: rationale and study protocol. BMC Cancer. (2016) 16:278. doi: 10.1186/s12885-016-2308-z, PMID: 27094927 PMC4837593

[27] Lenhart S Workman JT. Optimal control applied to biological models. Chapman Hall/CRC. (2007). doi: 10.1201/9781420011418

[28] Sharifi M Moridnia A Salehi R. Cancer immunotherapy by targeting immune checkpoints: Mechanism of T cell dysfunction and therapeutic approaches. J Biomed Sci. (2017) 24:35. doi: 10.1186/s12929-017-0341-0, PMID: 28545567 PMC5445514

[29] Dunn GP Bruce AT Ikeda H Old LJ Schreiber RD. Cancer immunoediting: From immunosurveillance to tumor escape. Nat Immunol. (2002) 3:991–8. doi: 10.1038/ni1102-991, PMID: 12407406

[30] Blank CU Haanen JB Ribas A Schumacher TN. The cancer immunogram. Science. (2016) 352:658–60. doi: 10.1126/science.aaf2834, PMID: 27151852

[31] Khajanchi S Nieto JJ. Mathematical modeling of tumor-immune competitive system, considering the role of time delay. Appl Math Comput. (2019) 340:180–205. doi: 10.1016/j.amc.2018.08.018

[32] Kuznetsov VA Makalkin IA Taylor MA Perelson AS. Nonlinear dynamics of immunogenic tumors: Parameter estimation and global bifurcation analysis. Bull Math Biol. (1994) 56:295–321. doi: 10.1007/BF02460644, PMID: 8186756

[33] Konstorum A Vella AT Adler AJ Laubenbacher RC. Addressing current challenges in cancer immunotherapy with mathematical and computational modelling. J R Soc Interface. (2017) 14:20170150. doi: 10.1098/rsif.2017.0150, PMID: 28659410 PMC5493798

[34] Manem VS Salgado R Aftimos P Sotiriou C Haibe-Kains B. Network science in clinical trials: A patient-centered approach. Clin Pharmacol Ther. (2018) 103:1008–11. doi: 10.1016/j.semcancer.2017.12.006, PMID: 29278737

[35] Navin NE. The first five years of single-cell cancer genomics and beyond. Genome Res. (2015) 25:1499–507. doi: 10.1101/gr.191098.115, PMID: 26430160 PMC4579335

[36] Siegel RL Miller KD Jemal A. Cancer statistics, 2021. CA. A Cancer J Clin. (2021) 71:7–33. doi: 10.3322/caac.21654, PMID: 33433946

[37] Welch HG Prorok PC O’Malley AJ Kramer BS. Breast-cancer tumor size, overdiagnosis, and mammography screening effectiveness. New Engl J Med. (2016) 375:1438–47. doi: 10.1056/NEJMoa1600249, PMID: 27732805

[38] Farrand KF Fridman M Stillman IÖ Schaumberg DA. Prevalence of diagnosed dry eye disease in the United States among adults aged 18 years and older. Am J Ophthalmol. (2017) 182:90–8. doi: 10.1016/j.ajo.2017.06.033, PMID: 28705660

[39] Rockne RC Hawkins-Daarud A Swanson KR Sluka JP Glazier JA Macklin P. The 2019 mathematical oncology roadmap. Phys Biol. (2019) 16:041005. doi: 10.1088/1478-3975/ab1a09, PMID: 30991381 PMC6655440

[40] Chakrabarti S Michor F. Pharmacokinetics and drug interactions determine optimum combination strategies in computational models of cancer evolution. Cancer Res. (2017) 77:3908–21. doi: 10.1158/0008-5472.CAN-16-2871, PMID: 28566331 PMC5553595

[41] Savas P Teo ZL Lefevre C Flensburg C Caramia F Alsop K. The subclonal architecture of metastatic breast cancer: Results from a prospective community-based rapid autopsy program “CASCADE. PloS Med. (2016) 13:e1002204. doi: 10.1371/journal.pmed.1002204, PMID: 28027312 PMC5189956

[42] Arafa AA Elnaggare MM. Mathematical modeling and optimal control for awareness and screening programs of breast cancer. Alexandria Eng J. (2020) 59:3893–903. doi: 10.1016/j.aej.2020.06.012

[43] Singh R Sarmah R Bhattacharyya D. Modeling and optimal control of breast cancer: Effects of chemotherapy and immunotherapy. Mathematics Comput Simulation. (2019) 162:118–37. doi: 10.1016/j.matcom.2018.04.012

[44] Fathoni M Gunardi G Kusumo FA Hutajulu SH. (2019). “Mathematical model analysis of breast cancer stages with side effects on heart in chemotherapy patients”, in: AIP Conference Proceedings, Yogyakarta, Indonesia, 29 July–1 August 2019. Melville, NY, USA: AIP Publishing. doi: 10.1063/1.5139153

[45] Deisboeck TS Wang Z Macklin P Cristini V. Multiscale cancer modeling. Annu Rev Biomed Eng. (2011) 13:127–55. doi: 10.1146/annurevbioeng071910124729 PMC3883359 21529163

[46] Werb D Rowell G Guyatt G Kerr T Montaner J Wood E. Effect of drug law enforcement on drug market violence: A systematic review. Int J Drug Policy. (2011) 22:87–94. doi: 10.1016/j.drugpo.2011.02.002, PMID: 21392957

[47] Benzekry S Lamont C Barbolosi D Hlatky L Hahnfeldt P. Mathematical modeling of tumor–tumor distant interactions supports a systemic control of tumor growth. Cancer Res. (2014) 74:2136–43. doi: 10.1158/0008-5472.CAN-13-2442, PMID: 28729417 PMC5600871

[48] Enderling H Park D Hlatky L Hahnfeldt P. The importance of spatial distribution of stemness and proliferation state in determining tumor radioresponse. Math Model Natural Phenomena. (2009) 4:117–33. doi: 10.1051/mmnp/20094405

[49] Pérez-García VM Calvo GF Bosque JJ Belmonte-Beitia J. A nonlinear model of tumor growth and cancer treatment with radiotherapy and chemotherapy. Math Biosci. (2011) 229:1–11. doi: 10.1016/j.mbs.2010.11.002, PMID: 21129385

[50] Dutta-Moscato J Kang Y Julien C Michailova A Myers J Marusyk A. A multiscale agent-based in silico model of ductal carcinoma in *situ* (DCIS) of the breast. IEEE Trans Biomed Eng. (2014) 61:707–18. doi: 10.1109/TBME.2013.2285410

[51] Macklin P Lowengrub JS. Nonlinear simulation of the effect of microenvironment on tumor growth. J Theor Biol. (2007) 245:677–704. doi: 10.1016/j.jtbi.2006.12.004, PMID: 17239903

[52] Dołęga-Kozierowski J Pędziwiatr M Pietka E Litniewski J. Numerical and physical modeling of breast cancer based on image fusion and artificial intelligence. Breast Cancer Res Treat. (2023) 200:335–48. doi: 10.1007/s10549-023-07056-1, PMID: 37490172 PMC10504219

[53] Peterson ML Yankeelov TE Weis JA. Novel computational biology modeling system can accurately forecast response to neoadjuvant therapy in early breast cancer. Breast Cancer Res. (2023) 25:54. doi: 10.1186/s13058-023-01654-z, PMID: 37165441 PMC10170712

[54] Li Y Thirumalai D. A mathematical model for phenotypic heterogeneity in breast cancer with implications for therapeutic strategies. J R Soc Interface. (2022) 19:20210803. doi: 10.1098/rsif.2021.0803, PMID: 35078336 PMC8790361

[55] Yang Y Gao H Zhang L Zhou Y Chen Y. Mathematical characterization of population dynamics in breast cancer cells treated with doxorubicin. Front Mol Biosci. (2022) 9:972146. doi: 10.3389/fmolb.2022.972146, PMID: 36172049 PMC9510895

[56] Zeng Q Chen C Chen C Song H Li M Yan J. Serum Raman spectroscopy combined with convolutional neural network for rapid diagnosis of HER2-positive and triple-negative breast cancer. Spectrochim Acta Part A: Mol Biomol Spectrosc. (2023) 286:122000. doi: 10.1016/j.saa.2022.122000, PMID: 36279798

[57] Pang J Ding N Liu X He X Zhou W Xie H. Prognostic value of the baseline systemic immune-inflammation index in HER2-positive metastatic breast cancer: exploratory analysis of two prospective trials. Ann Surg Oncol. (2025) 32:750–9. doi: 10.1245/s10434-024-16454-8, PMID: 39565489

[58] Liu X Zhang G Zhao L. Detection of transmembrane protein 100 in breast cancer: Correlation with Malignant progression and chemosensitivity. Cytojournal. (2024) 21:65. doi: 10.25259/Cytojournal_107_2024, PMID: 39917003 PMC11801651

[59] Zhu K Lv Q Qiao J. Efficacy and prognosis analysis of PD-1 inhibitor combined with GP regimen in the treatment of patients with advanced triple-negative breast cancer. Int J Pharmacol. (2024) 20:1030–9. doi: 10.3923/ijp.2024.1030.1039

[60] Cui N Wu J Zhang XC Pu YN Zhao B Han TT. Development and validation of a nomogram prediction model for moderate-to-severe acute radiation dermatitis in patients with breast cancer: A retrospective study. Br J Hosp Med. (2024) 85. doi: 10.12968/hmed.2024.0254, PMID: 39475022

[61] Chen W Liu Y Wang C Zhu J Li G Liu C. Cross-modal causal representation learning for radiology report generation. IEEE Trans Image Process. (2025) 34:2970–85. doi: 10.1109/TIP.2025.3568746, PMID: 40378020

[62] Michaelis L Menten ML. Die kinetik der invertinwirkung. Biochem Z. (1913) 49:333–69.

[63] de Pillis LG Radunskaya AE. The dynamics of an optimally controlled tumor model: A case study. Math Comput Model. (2001) 37:1221–44. doi: 10.1016/S0895-7177(03)90099-3

[64] Dinku B Tefera T. A mathematical model of tumor–immune and host cell interactions with chemotherapy and optimal control. J Mathematics. (2024) 2024:3395825. doi: 10.1155/2024/3395825

[65] Zhou H Singh P Alarcon T. Modelling and simulation of genotypic tumor mutational burden and phenotypic immunogenicity biomarkers in cancer immunoediting. Comput Syst Oncol. (2025) 2:45–62. doi: 10.1002/cso2.1012 39894008

[66] Chen Y Rahman MM Dutta A. Mathematical model of tumor immune microenvironment with application to combined PD-1/PD-L1 and IL-10 therapeutic antibodies. Math Biosci Eng. (2024) 21:865–87. doi: 10.3934/mbe.2024039, PMID: 38303449

[67] Zhou X Fan M Liu B. Modeling and dynamics of tumor-immune system with immunotherapy treatment. Comput Math Methods Med. (2017), 5684605. doi: 10.1155/2017/5684605

[68] Lakshmikantham V Deo SG. Stability Analysis of Nonlinear Systems. New York: Marcel Dekker (1991).

[69] Korobeinikov A Maini PK. Non-linear incidence and stability of infectious disease models. Math Med Biol: A J IMA. (2004) 21:113–28. doi: 10.1093/imammb/21.2.113 15778334

[70] Lakshmikantham V Leela S. Differential and integral inequalities: Theory and applications. New York, Academic Press (1981).

[71] Yang Y Li Y Li X. Modeling CAR-T cell immunotherapy with immune checkpoint inhibition in solid tumors. Sci Rep. (2020) 10:21816. doi: 10.1038/s41598-020-78786-7 33311592

[72] Arciero JC Jackson TL Kirschner DE. A mathematical model of tumor-immune evasion and siRNA treatment. Bull Math Biol. (2019) 81:4086–110. doi: 10.1007/s11538-019-00651-1

[73] Ozdemir BH Zhang Y Anderson ARA. Hybrid modeling of tumor–immune system interactions under treatment. PloS Comput Biol. (2022) 18:e1010323. doi: 10.1371/journal.pcbi.1010323, PMID: 35853038 PMC9337699

[74] Hirata Y Tada T Fujiwara H Kikuchi R Sugano K Shimada J. Modeling immune escape and equilibrium in tumor-immune dynamics. J Theor Biol. (2021) 523:110717. doi: 10.1016/j.jtbi.2021.110717, PMID: 33862089

[75] Perko L. Differential equations and Dynamical Systems. New York, Springer (2001).

[76] Edelstein-Keshet L. Mathematical Models in Biology. Philadelphia: SIAM (2005).

[77] Siegel RL Miller KD Jemal A. Cancer statistics, 2021. CA: A Cancer J Clin. (2021) 71:7–33. doi: 10.3322/caac.21654, PMID: 33433946

[78] Khalil HK. Nonlinear systems. 3rd. Upper Saddle River, NJ: Prentice Hall (2002).

[79] World Health Organization (WHO) International Agency for Research on Cancer (IARC). Breast cancer: Global cancer observatory, fact sheets and trends (2021). Available online at: https://gco.iarc.fr/today/data/factsheets/cancers/20-Breast-fact-sheet.pdf (Accessed August 4, 2025).

